# Effects of Progestin on Modulation of the Expression of Biomarkers in Endometriosis

**DOI:** 10.3390/biomedicines11072036

**Published:** 2023-07-20

**Authors:** Daniela Roxana Matasariu, Alexandra Irma Gabriela Bausic, Cristina Elena Mandici, Iuliana Elena Bujor, Alexandra Elena Cristofor, Elvira Bratila, Ludmila Lozneanu, Lucian Vasile Boiculese, Mihaela Grigore, Alexandra Ursache

**Affiliations:** 1 Department of Obstetrics and Gynecology, University of Medicine and Pharmacy “Gr. T. Popa”, 700115 Iasi, Romania; daniela.matasariu@umfiasi.ro (D.R.M.); iuliana-elena.bujor@d.umfiasi.ro (I.E.B.); alexandra-elena_s_mihaila@d.umfiasi.ro (A.E.C.); mihaela.grigore@umfiasi.ro (M.G.); alexandra.ursache@umfiasi.ro (A.U.); 2 Department of Obstetrics and Gynecology, “Cuza Vodă” Hospital, 700038 Iasi, Romania; 3 Department of Obstetrics and Gynecology, University of Medicine and Pharmacy “Carol Davila”, 020021 Bucharest, Romania; alexandra.bausic@drd.umfcd.ro (A.I.G.B.); elvira.bratila@umfcd.ro (E.B.); 4 Department of Obstetrics and Gynecology, “Prof. Dr. Panait Sîrbu” Obstetrics and Gynecology Hospital, 060251 Bucharest, Romania; 5 Department of Morpho-Functional Sciences I—Histology, University of Medicine and Pharmacy “Gr. T. Popa”, 700115 Iasi, Romania; 6 Biostatistics, Department of Preventive Medicine and Interdisciplinarity, University of Medicine and Pharmacy “Gr. T. Popa”, 700115 Iasi, Romania; lboiculese@gmail.com

**Keywords:** progestin, endometriosis, immunohistochemistry, osteopontin, CD44

## Abstract

Background: Our study aimed to examine the osteopontin (OPN) serum levels and tissue expression of CD44 and OPN in endometriosis-affected women both undergoing and not undergoing progestin treatment, and also to determine their involvement in the pathogenesis of endometriosis. Methods: Using an ELISA kit, we evaluated the OPN serum levels of healthy and endometriosis-affected women both undergoing and not undergoing progestin treatment. Immunohistochemical (IHC) analyses were used to assess the endometriotic tissue expressions of CD44 and OPN. Results: There were statistically significant higher OPN serum levels in the healthy control group compared to the women with endometriosis. Furthermore, there were higher OPN serum levels in the endometriosis-affected women undergoing the progestin treatment, but the difference did not reach statistical significance. In comparison to OPN, CD44 expression was significantly higher in all the endometriotic tissue glands and stroma, regardless of the patient’s treatment status. Compared to the group receiving therapy, the OPN levels were higher in the endometriosis group not receiving therapy. OPN’s robust cytoplasmic expression seemed to be associated with the non-treatment group. Conclusion: Endometriosis, CD44, and OPN appear to be closely related. This study suggests that endometriosis that has not been treated has an immunological profile distinct to endometriosis that has received treatment.

## 1. Introduction

Controversy still surrounds the pathogenesis of endometriosis, with its exact cause remaining elusive. The presence of endometrial glands and stroma outside the uterus is the main characteristic of this disease, which leads to symptoms such as pelvic pain, infertility, and menstrual irregularities [1].

There are several well-known hypotheses regarding the occurrence of endometriosis; the most accepted one relates to retrograde menstruation [2]. As revealing as it seems, and despite its wide acceptance, the retrograde menstruation theory fails to explain why the disease affects only 3.7% of the 90% of women who experience retrograde menstruation [3,4]. Thus, other pathogenic mechanisms, such as genetic, epigenetic, and immunological factors, must be involved in the development of the ectopic focuses of endometriosis, which can lead to changes in the microenvironment and immunological activity, in turn reshaping the normal signaling paths. Recently, scientists have been exploring various molecular factors that contribute to endometriosis development and progression [5,6,7,8].

Even though endometriosis is a benign condition, it has been shown that it has a number of malignant attributes, such as neo-angiogenesis, invasion, and metastasis [2,9]. Up to 40% of affected women will develop recurring endometriosis within a 5-year period, despite the fact that the condition is not lethal, and patients benefit from therapy. Furthermore, about 1% of patients develop a form of ovarian cancer due to the malignant transformation of lesions [5,10,11].

One of the most common phenotypes of endometriosis involves endometriotic cysts. Currently, in treating endometriosis, surgery, recommended only in women with persistent pain symptoms or intolerance to hormonal therapy, is used alongside with first-line medical treatment, but constant research on a potential molecular targeted treatment is being conducted. Although the exact etiology of this disease remains unknown, accumulating evidence suggests that dysregulated cell adhesion molecules and extracellular matrix interactions play a crucial role in its development and progression [2].

CD44 and OPN, known as secreted phosphoprotein 1 (SPP1), are known to interact and form a functional complex in various biological processes (for instance, in embryo attachment, activating immune and epithelial cells), including at the site of the endometrium. Both CD44 and OPN play important roles in tissue remodeling, the immune response, and cell adhesion. CD44 can bind to OPN, forming a complex that facilitates cell adhesion and migration [12].

CD44, a cell surface glycoprotein involved in cell adhesion and migration, has emerged as a promising focus for research seeking to understand the complex mechanism underlying endometriosis [1,2,13]. Studies have shown that CD44, among other adhesion proteins, may play an important role in this type of aberrant cell behavior (ectopic endometrial attachment to the peritoneum, cell migration, and implant survival due to altered apoptosis), with its modified expression being a constant finding in such studies. This multifunctional transmembrane glycoprotein is widely expressed in various cell types and tissues, being involved in numerous physiological (embryo attachment, activating immune and epithelial cells) and pathological processes (cell migration, proliferation, invasion, angiogenesis, and cell survival, involved in tumor progression) [2,5].

In vitro studies have shown that CD44 expression is increased in endometriotic tissues [14,15,16]. Torres et al. found that plasma concentrations of CD44 were significantly increased in endometriosis samples and were also significantly correlated with the presence of the disease, but, surprisingly, they did not find a correlation between these high concentrations and the clinic-pathological stage of the disease [17]. Additionally, other studies found that CD44 was highly expressed in the epithelial cells of the ectopic endometrium in in vivo rat models, compared to samples of the normal endometrium [18,19,20]. Specifically, CD44 is involved in peritoneal attachment, and its diverse functions are attributed to its ability to bind hyaluronan, a major component of the extracellular matrix, and to interact with other ligands, such as growth factors and cytokines [21]. Furthermore, the findings of an in vivo study using CD44 knockout mice models suggest that CD44 plays a fundamental role in the development of endometriotic lesions; it was found to affect cell functions such as adhesion, signaling, and migration [22].

It seems that in the normally located endometrial tissue of endometriosis-affected patients, CD44 has an up-regulated turnover. This may suggest that these patients are prone to ectopy and invasion. Also, studies have found a higher concentration of serum CD44 in these women when compared to unaffected women [22].

CD44 is implicated in the adhesion and invasion of endometrial cells outside the uterus. Enhanced CD44 expression facilitates the attachment of endometrial cells to the peritoneal surface and other sites, promoting the establishment of endometriotic lesions. In addition, this molecule is involved in the attachment of other cell types. Studies have reported that treatments with anti-CD44 antibody or hyaluronidase led to the decrease in the adherence of ovarian cancerous cells to the peritoneum. Moreover, hyaluronidase seems to be effective in decreasing the adherence of endometrial epithelial cells (EEC) and endometrial stromal cells (ESC) to peritoneal mesothelial cells (PMC) [23].

CD44 is also involved in modulating the immune response by interacting with immune cells, such as macrophages and T lymphocytes. CD44 expression on immune cells influences their recruitment to endometriotic lesions and promotes the release of inflammatory cytokines, perpetuating the inflammatory milieu [24].

Angiogenesis is crucial for the survival and growth of endometriotic lesions. CD44 has been implicated in angiogenesis due to its interaction with factors involved in blood vessel formation. CD44-expressing endometrial cells promote the release of angiogenic factors and facilitate the development of a vascular network necessary for lesion growth and maintenance. It is also worth mentioning that CD44 is involved in the self-renewal and differentiation of stem cells, with its altered expression and signaling suspected to influence the behavior of endometrial stem cells, thereby contributing to the pathogenesis of endometriosis [25].

OPN is a protein that is involved in various physiological and pathological processes, including inflammation, tissue remodeling, and immune regulation. In the context of endometriosis, OPN has been implicated in several aspects of the disease, although the exact molecular mechanism is yet not fully understood [26,27]. Konno et al. were the first researchers to study the function of OPN in endometriosis, discovering abundant levels of OPN in endometriosis tissue using immunohistostaining [28], which indicated that OPN was involved in the development of endometriosis [26]. OPN was also discovered to be a CD44 glycoprotein ligand, and cell–cell and cell–matrix interactions are hampered by the cell surface receptor CD44 [26,29].

Furthermore, OPN has been found to promote cell adhesion and invasion in endometriosis. It interacts with integrins and other cell surface receptors to facilitate the attachment and migration of endometrial cells to the peritoneal lining and other sites [26,30,31].

In endometriosis, OPN has been shown to play a role in modulating the immune response and promoting inflammation. It can recruit immune cells, such as macrophages and T cells, to the sites of endometriotic lesions, leading to the production of pro-inflammatory cytokines and chemokines [32,33].

Moreover, OPN promotes angiogenesis by stimulating the proliferation and migration of endothelial cells. It can interact with various growth factor signaling pathways involved in angiogenesis [34,35].

This study set out to assess the involvement of CD44 and OPN in endometriosis, as well as their potential use in novel treatment strategies for the disease. In this regard, our goal was to assess the expression of these two molecules and their unique characteristics in endometriotic tissue from progestin-treated and untreated women, in order to help guide future therapeutic research. We evaluated the immunoreactivity of the epithelial and stromal components in the endometriotic tissue to determine if CD44 and OPN expression levels were implicated in the progression of endometriosis (i.e., the extension of lesion fibrosis in endometriosis).

## 2. Materials and Methods

### 2.1. Patient Selection and Serum and Tissue Samples

Our observational study included 60 patients (18–42 years old) with stage III or IV endometriotic lesions; they were enrolled between January 2021 and January 2022. For the purpose of the study, endometriotic cyst tissue and serum were obtained from patients with endometriotic cysts who underwent surgery at the Obstetrics and Gynecology Hospital “Cuza-Voda” in Iasi and the Obstetrics and Gynecology Hospital “Panait Sirbu” in Bucharest.

Serum samples were collected from the 60 endometriosis-positive women involved in the study, with 24 of the patients undergoing 2 mg daily dienogest treatment for 3 months prior to surgery, and the other 36 not undergoing any treatment. Serum sampling was performed twice: on the day of the procedure (M2) and three months before the intervention (M1), when they were clinically and para-clinically identified to have endometriosis. The serum samples from the M1 time frame were compared to the OPN serum levels of 30 controls who met our exclusion criteria, and whose age and body mass index (BMI) values matched those of our study group. The women from the control group attended the two hospitals for laparoscopic tubal ligation without any gynecological complaints, and there were no intraoperative incidental endometriosis findings.

We also collected endometriotic tissue specimens from the 60 endometriosis-positive women; as mentioned above, 24 were undergoing hormonal treatment while 36 were not undergoing any treatment.

All patients provided written informed consent before being enrolled in the study, and the Hospital Ethics Committee approved the study (No. 11062/2020 and No. 6/2021).

#### 2.1.1. Inclusion Criteria

We obtained serum and tissue samples from female patients whose endometriosis had been laparoscopically identified and later histologically confirmed. Specifically, tissue samples were collected from endometriotic ovarian cysts of women who had undergone surgery and who had and had not undergone hormone therapy with progestins (dienogest 2 mg daily) for three months prior to the intervention.

#### 2.1.2. Exclusion Criteria

Due to the possibility that these factors could affect our research, we excluded women with BMI > 30, malignant or other tumoral lesions, diabetes, depression, genetic syndromes, any infectious or autoimmune diseases, as well as who smoked, were pregnant, were taking any other hormonal therapy besides dienogest, or were undergoing any other treatment that might interfere with bone and mineral metabolisms.

Histomorphological and immunohistochemistry (IHC) testings confirmed the pathological diagnosis of endometriosis in every case. Routinely prepared hematoxylin eosin (H&E) sections were examined, and IHC was independently evaluated by two pathologists to confirm the diagnosis.

### 2.2. Enzyme-Linked Immunosorbent Assay (ELISA)

Using the manufacturer’s instructions, an enzyme-linked immunosorbent assay (ELISA) kit (Reference DY1433 Human OPN; R&D Systems, Inc., Minneapolis, MN, USA) was used to quantify OPN levels in serum. The measurement range was 30 to 4000 mg/L. OPN concentrations less than 30 mg/L were not detectable and were considered as 0 mg/L for statistical purposes. Every sample was examined twice, and the mean value was computed. The OPN ELISA kit’s intra- and inter-assay coefficient variability values were 7.8% and 9.8%, respectively.

### 2.3. Immunohistochemistry

Immunohistochemistry was carried out on formalin-fixed, paraffin-embedded tissue utilizing monoclonal antibodies against CD44 and OPN. The samples were fixed with 10% neutral formalin before being paraffin embedded and cut into sections that were 4–5 μm thick. IHC was used to determine the expressions of CD44 and OPN, with specific dilutions used for CD44 (1:250) and OPN (1:200), as suggested by the provider (Abcam, Cambridge, UK) (Table 1).

CD44 expression was primarily found at the membrane level, whereas OPN was predominantly located in the cytoplasm of the epithelium and stroma of endometriotic cells. The distribution of CD44 in the endometriotic tissues was homogeneous, in contrast to OPN, whose distribution was heterogeneous.

The intensity (0—negative, +1 weak, +2 moderate, +3 strong) and percentage of positive cells (0–100%) were analyzed and categorized into two groups showing negative (negative and only focal weak intensity in < 10% cells) and positive scores (moderate to strong intensity in >10% cells), respectively.

### 2.4. Statistical Analysis

Medical data were imported into and verified in Microsoft Excel and then analyzed in SPSS 24 (IBM Corp. Released 2016. IBM SPSS Statistics for Windows, Version 24.0. Armonk, NY, USA: IBM Corp.).

The data were in the form of measurable numerical values on a ratio scale or categorical variables on an interval scale. Within the descriptive statistics, we calculated the values of the following statistical measures: sample size (N), mean, standard deviation, standard error, and 95% confidence intervals for mean, min, max, absolute, and relative frequencies.

Statistical hypothesis tests were conducted using the Student’s *t*-test (for comparing the averages of numerical variables), the Levene’s test (for checking the equality of variances), and chi-square or Fisher’s exact test (for categorical type).

The standard cut-off of 5% or 0.05 significance was used to determine whether the hypothesis was supported.

## 3. Results

The mean age of the 60 women with endometriosis (24 who were undergoing treatment and 36 who were not) was 31.92 ± 4.706 years (30.70/33.13—95% CI).

### 3.1. Serum OPN Levels

For the M1 time frame, we found statistically significant higher OPN levels in the patients from the control group (without endometriosis detected during laparoscopic surgery) compared to the 60 women with endometriotic ovarian cysts (Table 2).

At the M2 time frame, when we compared the mean serum OPN levels of the 24 endometriosis patients receiving progestin therapy with the 36 endometriosis patients receiving no specific endometriosis treatment, the treated women were found to have higher levels, but the difference was not statistically significant (Table 2).

### 3.2. Evaluation of CD44 and OPN Endometriotic Sample Expression

All cases of endometriosis, with and without treatment, showed an intensely positive reaction for CD44. Despite the fact that CD44 was substantially expressed in every case of endometriosis, a positive score was discovered in 32 (60%) of the untreated cases and 21 (40%) of the treated cases. OPN was expressed positively in 28 cases: 14 cases (50%) in the non-treatment group and 14 cases (50%) in the treatment group (Table 3). OPN exhibited higher positive expression in the treatment group, recording 14 positive (50%) and 10 (31.25%) negative tissue samples in this group. In the specimens from the women who were not undergoing progestin treatment, the tissue OPN expression was positive in 14 (50%) and negative in 22 (68.75%) samples (Table 3).

**Figure 1:**
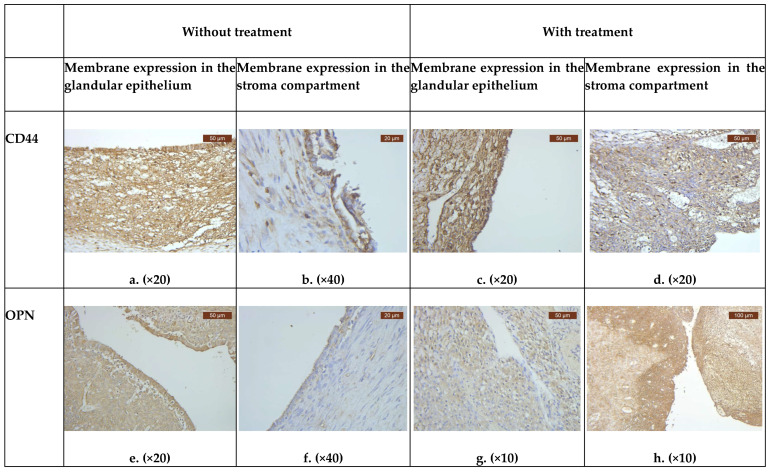
Immunohistochemical expression of CD44 and OPN in two groups (with and without treatment).

According to the immunohistochemistry results, both the stroma and endometriotic cells highly expressed CD44 (Figure 1a–d). OPN was mostly expressed in the stroma and showed a comparable pattern of expression in both the treatment- and without treatment-groups (Figure 1e–h).

When we examined the CD44 and tissue OPN expression, there was no statistically significant difference between the progestin-treated and without-treatment groups, with *p* values of 0.99 and 0.14 obtained, respectively (Table 3).

However, when we separately analyzed the epithelial and stromal compartments, we detected some intriguing findings regarding OPN’s expression. A total of 20 (55%) of the endometriotic areas in the group who received no therapy showed a negative or only focal and weak reaction for OPN, while 16 (45%) of the remaining areas in that group showed a positive immunoreaction for OPN with a moderate to strong intensity. While in the treatment group the distribution of OPN was comparable to the non-treatment group, the lack of progestin treatment seemed to correlate with higher OPN expression in the epithelial compartment and with low expression in the stromal compartment (Table 4). When studying the CD44 separate compartments, this pattern was absent.

It seems that a positive correlation between the tissue OPN and serum OPN was present.

## 4. Discussion

The search for markers of and potential treatments for endometriosis is ongoing. Due to the still poorly understood condition’s pathophysiology, there are just a few options for treatments with less severe side effects, mostly progestin therapies and medication for alleviating pain. There are currently no new drugs available for the treatment of the disease, partly because the underlying causes of endometriosis are still unclear. Progestin therapy has been used alone or in conjunction with other medications to treat endometriosis [19,36,37,38,39].

In this study, we investigated CD44 and OPN, two molecules thought to be important in the onset and progression of endometriosis, to determine if they exhibit any changes in response to progestin therapy. The results of progestin therapy vary from patient to patient, are effective in about two-thirds of patients [40,41], and have a limited long-term impact [42,43,44]. Additionally, some recent studies have reported response specificities in ectopic and eutopic endometrial tissue, as well as that eutopic endometrial tissue exhibits a distinct response to progesterone in both healthy and endometriosis-affected women [19]. According to Guidice et al. [45], the expression of solely progesterone receptor inhibitory isoform A causes endometriotic implants to be resistant to progesterone.

In the pathophysiology of endometriosis, CD44 and OPN seem to both play some important role by influencing a multitude of underlying processes that result in the appearance and growth of endometriotic ectopic tissue, such as adhesion, invasion, inflammation, angiogenesis, and stem cell activity. Understanding how these two molecules are involved in endometriosis offers important insights into the underlying disease process and may provide novel diagnostic and treatment options. There is a lack of data concerning the levels of OPN in women with endometriosis and the levels of CD44 and OPN in endometriotic ectopic tissue from endometriosis specimens [26,46,47,48].

Studies have reported conflicting results regarding serum OPN levels in women with endometriosis compared to controls. OPN is a protein involved in various physiological processes, including inflammation, immune response, and tissue remodeling. Some studies have suggested that serum OPN levels are higher in endometriosis patients compared to controls, while others have found no significant difference or even lower levels in endometriosis patients. Studies by Ho et al., Fu et al., D’Amico et al., and Cho at al., published in 2009, 2013, 2021, and 2022, respectively, found elevated serum OPN levels in women with endometriosis [26,34,49,50]. On the other hand, Streuli et al. found lower OPN serum levels in women with focal adenomyosis compared to controls and in women with focal adenomyosis and deep infiltrating endometriosis. The patients included in this 2017 study had either diffuse or focal adenomyosis and one of the three types of endometriosis: superficial, deep infiltrating, or endometriosis cysts. Thus, even though the results reported by Streuli et al. are comparable with ours, it is difficult to draw a comparison [51].

There is limited research that specifically compares CD44 endometriotic tissue staining in endometriosis-affected women who have and have not undergone progestin treatment [2,46,52,53]. In 2007, Kim et al. studied the expression of this marker in tissue from various phenotypic types of endometriosis (peritoneal, ovarian, and rectovaginal). The results of their immunohistochemistry analysis showed that CD44 was mostly expressed in the stroma compartment of endometriotic ectopic lesions, with consistent expression of this molecule found in both the epithelial and stromal compartments [52]. In 2013, Koo et al. evaluated the tissue levels and circulating levels of CD44 in women with endometriosis. The authors performed an endometrial stromal cell (ESC) to peritoneal mesothelial cell (PMC) adhesion assay in women with and without endometriosis. They evaluated the levels of the CD44 mRNA in the endometrial stromal cells of women with and without endometriosis using reverse transcription-polymerase chain reaction (rPCR), and the serum CD44 levels in the same women using the western blot technique. They found no statistically significant differences between the expression of CD44 in the ESCs of the women with and without endometriosis, with no differences in attachment also found, even after CD44 antibody treatment, indicating that this molecule may not be directly involved in the attachment of ESC to PMC in women with this pathology, an attachment that serves as the foundation for endometriosis appearance [46]. A 2015 animal study by Knudtson et al. utilizing knockout mice highlighted the role of this molecule in the early stages of endometriosis development, although they found that this marker was not the only one involved, because endometriotic lesions still manifested in the absence of CD44 in the knockout mice [53]. A more recent, 2020 study, that evaluated CD44 expression in the endometrium and ectopic endometriotic lesions of women with and without endometriosis, surprisingly found lower levels of this cell-surface glycoprotein in the women with endometriosis compared to controls, with CD44 IHC analysis suggesting that it was mostly localized in the cytoplasm of glandular and stromal cells [2]. Moreover, Pazhohan et al. and Matsuzaki et al. found higher CD44 expression in the eutopic and/or ectopic endometrial samples of women with endometriosis compared to controls [22,54], a finding in opposition to that of Poncelet at al. and Nothnick et al., who both found lower levels in women with endometriosis than in controls [1,55]. Our research went beyond these previous studies. We wanted to establish whether progestin therapy influences the expression of these markers in endometriosis-affected women. We found that women with endometriosis who were not receiving progestin therapy had increased levels of CD44 expression in both stromal and epithelial compartments. Even though this did not approach statistical significance, we still think it is an extremely important finding for future research, because the tissue samples in the study were collected just three months after the patients started progestin therapy. In endometriosis specimens, reduced CD44 tissue levels indicate a quantifiable therapeutic response.

The effects of progestins on CD44 expression in endometriosis may depend on various factors, including the specific dosage, treatment duration, and patient’s individual characteristics. Similar to our study, some studies suggest that progestins may decrease CD44 expression in endometriotic lesions, but the exact impact of this and its extent require further investigation [2,46,52,53].

To the best of our knowledge, there are no specific studies to date that directly compare tissue OPN staining levels between progestin-treated women and untreated women with endometriosis. Fu et al.’s study found that OPN was upregulated in endometriotic cellular cultures from participants with endometriosis [34]. A 2021 study using an advanced preclinical model of endometriosis found that OPN expression levels in both eutopic and ectopic endometriosis organoids were statistically significantly lower than its expression levels in control endometrial organoids [34,56]. Additionally, Tremaine et al. found in their IHC investigation of the ovine endometrium that progesterone therapy increased OPN levels [57]. Paravati et al. evaluated both markers in PCOS patients with and without ovulation. They suggested there was a connection between both the serum levels and tissue expression of CD44 and OPN in the PCOS-affected women [12]. Our results support Paravati et al.’s finding that these two markers are directly associated, even though their study only included PCOS-positive women, not women with endometriosis [12]. The findings of our investigation showed a less pronounced alteration in tissue OPN expression than what was observed in the CD44 case.

Our results suggest that endometriotic ovarian cyst specimens exhibit decreased expression of CD44 and OPN in both epithelium and stroma compartments after progestin therapy. Our results provide evidence for the beneficial molecular effects of progestin therapy in endometriosis-affected women and the close relationship between these two markers. For OPN to be expressed on the endometrial surface, OPN’s receptor CD44 must be expressed on the endometrial epithelial cell membrane. According to Cao et al. and D’Amico et al., but contrary to Nothnick at al., decreased expression of these molecules is linked to cancer metastasis, tumor invasion, cancer cell proliferation, cell migration, and chemotherapy resistance in a variety of cells and tissues [30,49,55].

Despite the scarce data in the literature, contradictory results in this research area have emerged. We need to take into consideration the fact that such results may differ depending on the study design, sample size, demographic characteristics, and assay technique variations. More research in this field is needed to determine the precise association between endometriosis and the serum and tissue levels of OPN and CD44 expression. Blood biomarkers will always represent a tempting, less intrusive option to use as part of paraclinical investigations in women with endometriosis. Because of the parallel between the tissue and blood values found in this study, the use of blood biomarkers may seem like a feasible option, but further research on women with endometriosis is required to confirm this.

Our research has some limitations. Firstly, the study’s small sample size and limited timeframe may have had an impact on our findings. To corroborate these, we need to conduct additional research on a broader population. Secondly, every woman in our study had an ovarian endometriotic cyst, a form of phenotypically expressed endometriosis, and was in stage III or IV of the disease. We must consider all the disease’s stages in all of its phenotypic manifestations in order to validate and generalize the findings of the research. Despite this, we are optimistic that our findings may spur additional research into the etiology and pathogeny of endometriosis, as well as the development of accurate, noninvasive diagnostic methods, and better treatment options for the condition.

## 5. Conclusions

This research provides insight into a strong relationship between OPN, CD44, and endometriosis. Additionally, we noticed that progestin significantly impacted these tissue markers that are involved in cell growth and apoptosis.

In this study, we found that healthy women had greater OPN serum levels than endometriosis patients both undergoing and not undergoing therapy. After just three months of progestin therapy, a rise in this marker’s blood levels was detected.

In the endometriosis-affected women, progesterone therapy appeared to alter the expression of these two markers in the tissue. Higher OPN expression in the epithelial compartment and lower expression in the stromal compartment appeared to be correlated with a lack of progestin therapy. This pattern was not found in the CD44 distinct compartment investigation, where CD44 was found to be highly responsive in all cases, i.e., in patients both undergoing and not undergoing therapy.

In conclusion, our study revealed that progestin can be used to influence the progression of endometriosis by reducing proliferation and migration of the endometriotic cells.

## Figures and Tables

**Table 1:** Immunohistochemical panel of antibodies used in the study.

Antibody	Clone, Manufacturer	Dilution	Expression
Anti—CD44	Rabbit polyclonal IgG isotype, Abcam (ab157107)	1:250	Nuclear
Anti—OPN	Rabbit polyclonal IgG isotype, Abcam (ab8448)	1:200	Nuclear

OPN: osteopontin; IgG: immunoglobulin G.

**Table 2:** Serum OPN levels in women with endometriosis who were and were not undergoing treatment.

Group	N	Mean	Std. Deviation	Std Error	95% Confidence Interval for Mean		Min	Max	*p*
					Lower Bound	Upper Bound			
M1 before treatment									<0.01
endometriosis group	60	210.061	219.088	28.284	153.465/266.658		74	1070.92	
control group	30	390.101	303.458	55.403	276.788/503.414		80.92	1086	
M2									0.053
without treatment	36	201.918	204.253	34.042	132.809/271.028		74	1070.92	
with treatment	24	318.943	235.269	48.024	219.597/418.289		75.20	893.810	

**Table 3:** Differential expression of CD44 and OPN in endometriotic samples.

Group	N	CD44		OPN	
		Positive	Negative	Positive	Negative
With treatment	24	21 (40%)	3 (43%)	14 (50%)	10 (31.25%)
Without treatment	36	32 (60%)	4 (57%)	14 (50%)	22 (68.75%)
*p*-value		0.99 F		0.14	

F—Fisher exact test.

**Table 4:** Differential expression levels of CD44 and OPN in endometriotic samples in the two compartments.

		CD44				OPN			
Group	N	Epithelial		Stromal		Epithelial		Stromal	
		Positive	Negative	Positive	Negative	Positive	Negative	Positive	Negative
With treatment	24	22 (39.28%)	2 (50%)	17 (38.63%)	7 (43.75%)	14 (41.17%)	10 (38.46%)	13 (14.81%)	11 (33.33%)
Without treatment	36	34 (60.71%)	2 (50%)	27 (61.36%)	9 (56.25%)	20 (58.82%)	16 (61.53%)	14 (51.85%)	22 (66.66%)
*p*-value		56	4	44	16	34	26	27	33
		0.99 F		0.72		0.83		0.24	

F—Fisher exact test.

## Data Availability

The data used to support the findings of this study are available upon request to the corresponding author.

## References

[1] Poncelet C., Leblanc M., Walker-Combrouze F., Soriano D., Feldmann G., Madelenat P., Scoazec J.Y., Daraï E. (2002). Expression of cadherins and CD44 isoforms in human endometrium and peritoneal endometriosis. Acta Obstet. Gynecol. Scand..

[2] Sancakli Usta C., Turan G., Bulbul C.B., Usta A., Adali E. (2020). Differential expression of Oct-4, CD44, and E-cadherin in eutopic and ectopic endometrium in ovarian endometriomas and their correlations with clinicopathological variables. Reprod. Biol. Endocrinol..

[3] Mignemi G., Facchini C., Raimondo D., Montanari G., Ferrini G., Seracchioli R. (2012). A case report of nasal endometriosis in a patient affected by Behcet’s disease. J. Minim. Invasive Gynecol..

[4] Sourial S., Tempest N., Hapangama D.K. (2014). Theories on the pathogenesis of endometriosis. Int. J. Reprod. Med..

[5] Zhao M., Zhang M., Yu Q., Fei W., Li T., Zhu L., Yao Y., Zheng C., Zhang X. (2022). Hyaluronic Acid-Modified Nanoplatforms as a Vector for Targeted Delivery of Autophagy-Related Gene to the Endometriotic Lesions in Mice. Front. Bioeng. Biotechnol..

[6] Gadducci A., Multinu F., Cosio S., Carinelli S., Ghioni M., Aletti G.D. (2021). Clear cell carcinoma of the ovary: Epidemiology, pathological and biological features, treatment options and clinical outcomes. Gynecol. Oncol..

[7] Lin S., Xie X., Guo Y., Zhang H., Liu C., Yi J., Su Y., Deng Q., Zhu W. (2020). Clinical characteristics and pregnancy outcomes of infertile patients with endometriosis and endometrial polyps: A retrospective cohort study. Taiwan. J. Obstet. Gynecol..

[8] Mehdizadehkashi A., Tahermanesh K., Fazel Anvari-Yazdi A., Chaichian S., Azarpira N., Nobakht M., Abed S.M., Hashemi N. (2017). Ultrastructural Investigation of Pelvic Peritoneum in Patients with Chronic Pelvic Pain and Subtle Endometriosis in Association with Chromoendoscopy. J. Minim. Invasive Gynecol..

[9] Siufi Neto J., Kho R.M., Siufi D.F., Baracat E.C., Anderson K.S., Abrão M.S. (2014). Cellular, histologic, and molecular changes associated with endometriosis and ovarian cancer. J. Minim. Invasive Gynecol..

[10] Kurose S., Nakayama K., Razia S., Ishikawa M., Ishibashi T., Yamashita H., Sato S., Sakiyama A., Yoshioka S., Kobayashi M. (2021). Whole-Exome Sequencing of Rare Site Endometriosis-Associated Cancer. Diseases.

[11] Mechsner S. (2016). Endometriose: Eine oft verkannte Schmerzerkrankung [Endometriosis: An often unrecognized pain disorder]. Schmerz.

[12] Paravati R., De Mello N., Onyido E.K., Francis L.W., Brüsehafer K., Younas K., Spencer-Harty S., Conlan R.S., Gonzalez D., Margarit L. (2020). Differential regulation of osteopontin and CD44 correlates with infertility status in PCOS patients. J. Mol. Med..

[13] Kiyama R. (2020). Nutritional implications of ginger: Chemistry, biological activities and signaling pathways. J. Nutr. Biochem..

[14] Wojciechowski M., Krawczyk T., Śmigielski J., Malinowski A. (2015). CD44 expression in curettage and postoperative specimens of endometrial cancer. Arch. Gynecol. Obstet..

[15] Elbasateeny S.S., Salem A.A., Abdelsalam W.A., Salem R.A. (2016). Immunohistochemical expression of cancer stem cell related markers CD44 and CD133 in endometrial cancer. Pathol. Res. Pract..

[16] El-Sahwi K., Bellone S., Cocco E., Casagrande F., Bellone M., Abu-Khalaf M., Buza N., Tavassoli F.A., Hui P., Rüttinger D. (2010). Overexpression of EpCAM in uterine serous papillary carcinoma: Implications for EpCAM-specific immunotherapy with human monoclonal antibody adecatumumab (MT201). Mol. Cancer Ther..

[17] Torres A., Pac-Sosińska M., Wiktor K., Paszkowski T., Maciejewski R., Torres K. (2019). CD44, TGM2 and EpCAM as novel plasma markers in endometrial cancer diagnosis. BMC Cancer.

[18] Zhao M.D., Cheng J.L., Yan J.J., Chen F.Y., Sheng J.Z., Sun D.L., Chen J., Miao J., Zhang R.J., Zheng C.H. (2016). Hyaluronic acid reagent functional chitosan-PEI conjugate with AQP2-siRNA suppressed endometriotic lesion formation. Int. J. Nanomed..

[19] Iwase A., Kotani T., Goto M., Kobayashi H., Takikawa S., Nakahara T., Nakamura T., Kondo M., Bayasula, Nagatomo Y. (2014). Possible involvement of CD10 in the development of endometriosis due to its inhibitory effects on CD44-dependent cell adhesion. Reprod. Sci..

[20] Heidari-Keshel S., Rezaei-Tavirani M., Ai J., Soleimani M., Baradaran-Rafii A., Ebrahimi M., Roozafzoon R., Rahmanzadeh S., Raeisossadati R., Omidi R. (2015). Tissue-specific somatic stem-cell isolation and characterization from human endometriosis. Key roles in the initiation of endometrial proliferative disorders. Minerva Med..

[21] Niiro E., Kawahara N., Yamada Y., Yoshimoto C., Shimada K., Sudo T., Kobayashi H. (2019). Immunohistochemical expression of CD44v9 and 8-OHdG in ovarian endometrioma and the benign endometriotic lesions adjacent to clear cell carcinoma. J. Obstet. Gynaecol. Res..

[22] Pazhohan A., Amidi F., Akbari-Asbagh F., Seyedrezazadeh E., Aftabi Y., Abdolalizadeh J., Khodarahmian M., Khanlarkhani N., Sobhani A. (2018). Expression and shedding of CD44 in the endometrium of women with endometriosis and modulating effects of vitamin D: A randomized exploratory trial. J. Steroid Biochem. Mol. Biol..

[23] Olivares C.N., Alaniz L.D., Menger M.D., Barañao R.I., Laschke M.W., Meresman G.F. (2016). Inhibition of Hyaluronic Acid Synthesis Suppresses Angiogenesis in Developing Endometriotic Lesions. PLoS ONE.

[24] Hou W., Kong L., Hou Z., Ji H. (2022). CD44 is a prognostic biomarker and correlated with immune infiltrates in gastric cancer. BMC Med. Genom..

[25] Chen L., Fu C., Zhang Q., He C., Zhang F., Wei Q. (2020). The role of CD44 in pathological angiogenesis. FASEB J..

[26] Ho N.T., Lin S.W., Lee Y.R., Tzeng C.R., Kao S.H. (2022). Osteopontin Splicing Isoforms Contribute to Endometriotic Proliferation, Migration, and Epithelial-Mesenchymal Transition in Endometrial Epithelial Cells. Int. J. Mol. Sci..

[27] Wang W., Li P.N., Li W., Jiang J., Cui Y., Li S., Wang Z. (2017). Osteopontin activates mesenchymal stem cells to repair skin wound. PLoS ONE.

[28] Konno R., Fujiwara H., Netsu S., Odagiri K., Shimane M., Nomura H., Suzuki M. (2007). Gene Expression Profiling of the Rat Endometriosis Model. Am. J. Reprod. Immunol..

[29] Goodison S., Urquidi V., Tarin D. (1999). CD44 cell adhesion molecules. Mol. Pathol..

[30] Zhao H., Chen Q., Alam A., Cui J., Suen K.C., Soo A.P., Eguchi S., Gu J., Ma D. (2018). The role of osteopontin in the progression of solid organ tumour. Cell Death Dis..

[31] Cao Y., Liu X., Guo S.W. (2019). Plasma High Mobility Group Box 1 (HMGB1), Osteopontin (OPN), and Hyaluronic Acid (HA) as Admissible Biomarkers for Endometriosis. Sci. Rep..

[32] Lin E.Y., Xi W., Aggarwal N., Shinohara M.L. (2023). Osteopontin (OPN)/SPP1: From its biochemistry to biological functions in the innate immune system and the central nervous system (CNS). Int. Immunol..

[33] Rittling S.R., Singh R. (2015). Osteopontin in Immune-mediated Diseases. J. Dent. Res..

[34] Fu X., Yao M., Ye C., Fang T., Wu R. (2021). Osteopontin Regulates Endometrial Stromal Cell Migration in Endometriosis through the PI3K Pathway: Osteopontin Regulates Endometrial Cell Migration in Endometriosis. Reprod. Sci..

[35] Subraman V., Thiyagarajan M., Malathi N., Rajan S.T. (2015). OPN—Revisited. J. Clin. Diagn. Res..

[36] Allaire C., Bedaiwy M.A., Yong P.J. (2023). Diagnosis and management of endometriosis. CMAJ.

[37] Bouquet de Joliniere J., Fruscalzo A., Khomsi F., Stochino Loi E., Cherbanyk F., Ayoubi J.M., Feki A. (2021). Antiangiogenic Therapy as a New Strategy in the Treatment of Endometriosis? The First Case Report. Front. Surg..

[38] Malvezzi H., Marengo E.B., Podgaec S., de Azevedo Piccinato C. (2020). Endometriosis: Current challenges in modeling a multifactorial disease of unknown etiology. J. Transl. Med..

[39] Brichant G., Laraki I., Henry L., Munaut C., Nisolle M. (2021). New Therapeutics in Endometriosis: A Review of Hormonal, Non-Hormonal, and Non-Coding RNA Treatments. Int. J. Mol. Sci..

[40] Vercellini P., Buggio L., Berlanda N., Barbara G., Somigliana E., Bosari S. (2016). Estrogen-progestins and progestins for the management of endometriosis. Fertil. Steril..

[41] Vercellini P., Buggio L., Frattaruolo M.P., Borghi A., Dridi D., Somigliana E. (2018). Medical treatment of endometriosis-related pain. Best Pract. Res. Clin. Obstet. Gynaecol..

[42] Surrey E.S., Soliman A.M., Johns B., Vora J.B., Taylor H.S., Agarwal S.K. (2020). Real-World Characterization of Women with Diagnosed Endometriosis Initiating Therapy with Elagolix Using a US Claims Database. Clin. Outcomes Res..

[43] Soliman A.M., Coyne K.S., Zaiser E., Castelli-Haley J., Fuldeore M.J. (2017). The burden of endometriosis symptoms on health-related quality of life in women in the United States: A cross-sectional study. J. Psychosom. Obstet. Gynecol..

[44] Soliman A.M., Yang H., Du E.X., Kelley C., Winkel C. (2016). The direct and indirect costs associated with endometriosis: A systematic literature review. Hum. Reprod..

[45] Giudice L.C., Kao L.C. (2004). *Endometriosis*. Lancet.

[46] Koo Y.H., Na Y.J., Ahn M.Y., Jeon H.N., Yeom J.I., Lee K.S. (2013). Expression of CD44 in endometrial stromal cells from women with and without endometriosis and its effect on the adherence to peritoneal mesothelial cells. Obstet. Gynecol. Sci..

[47] Yang M., Jiang C., Chen H., Nian Y., Bai Z., Ha C. (2015). The involvement of osteopontin and matrix metalloproteinase- 9 in the migration of endometrial epithelial cells in patients with endometriosis. Reprod. Biol. Endocrinol..

[48] Nisenblat V., Bossuyt P.M., Shaikh R., Farquhar C., Jordan V., Scheffers C.S., Mol B.W., Johnson N., Hull M.L. (2016). Blood biomarkers for the non-invasive diagnosis of endometriosis. Cochrane Database Syst. Rev..

[49] D’Amico F., Skarmoutsou E., Quaderno G., Malaponte G., La Corte C., Scibilia G., D’Agate G., Scollo P., Fraggetta F., Spandidos D.A. (2013). Expression and localisation of osteopontin and prominin-1 (CD133) in patients with endometriosis. Int. J. Mol. Med..

[50] Cho S., Ahn Y.S., Choi Y.S., Seo S.K., Nam A., Kim H.Y., Kim J.H., Park K.H., Cho D.J., Lee B.S. (2009). Endometrial osteopontin mRNA expression and plasma osteopontin levels are increased in patients with endometriosis. Am. J. Reprod. Immunol..

[51] Streuli I., Santulli P., Chouzenoux S., Chapron C., Batteux F. (2017). Serum Osteopontin Levels Are Decreased in Focal Adenomyosis. Reprod. Sci..

[52] Kim H.O., Yang K.M., Kang I.S., Koong M.K., Kim H.S., Zhang X., Kim I. (2007). Expression of CD44s, vascular endothelial growth factor, matrix metalloproteinase-2 and Ki-67 in peritoneal, rectovaginal and ovarian endometriosis. J. Reprod. Med..

[53] Knudtson J.F., Tekmal R.R., Santos M.T., Binkley P.A., Krishnegowda N., Valente P., Schenken R.S. (2016). Impaired Development of Early Endometriotic Lesions in CD44 Knockout Mice. Reprod. Sci..

[54] Matsuzaki S., Darcha C., Maleysson E., Canis M., Mage G. (2010). Impaired down-regulation of E-cadherin and beta-catenin protein expression in endometrial epithelial cells in the mid-secretory endometrium of infertile patients with endometriosis. J. Clin. Endocrinol. Metab..

[55] Nothnick W.B., Fan F., Iczkowski K.A., Ashwell R., Thomas P., Tawfik O.W. (2001). CD44s expression is reduced in endometriotic lesions compared to eutopic endometrium in women with endometriosis. Int. J. Gynecol. Pathol..

[56] Esfandiari F., Heidari Khoei H., Saber M., Favaedi R., Piryaei A., Moini A., Shahhoseini M., Ramezanali F., Ghaffari F., Baharvand H. (2021). Disturbed progesterone signalling in an advanced preclinical model of endometriosis. Reprod. Biomed. Online.

[57] Tremaine T.D., Fouladi-Nashta A.A. (2021). Steroid regulation of secreted phosphoprotein 1 (SPP1) expression in ovine endometrium. Reprod. Fertil. Dev..

